# Supplementary material for: Identification of Muscle Strength-Related Gut Microbes through Human Fecal Microbiome Transplantation
Source: Int J Mol Sci. 2024 Jan 4;25(1):662. doi: 10.3390/ijms25010662 (PMC10779158; doi:10.3390/ijms25010662)
Supplement: Supplementary file 1 [file ijms-25-00662-s001.zip › ijms-2777162-supplementary.pdf]

Supplementary Information

Supplemental Figures

Figure S1

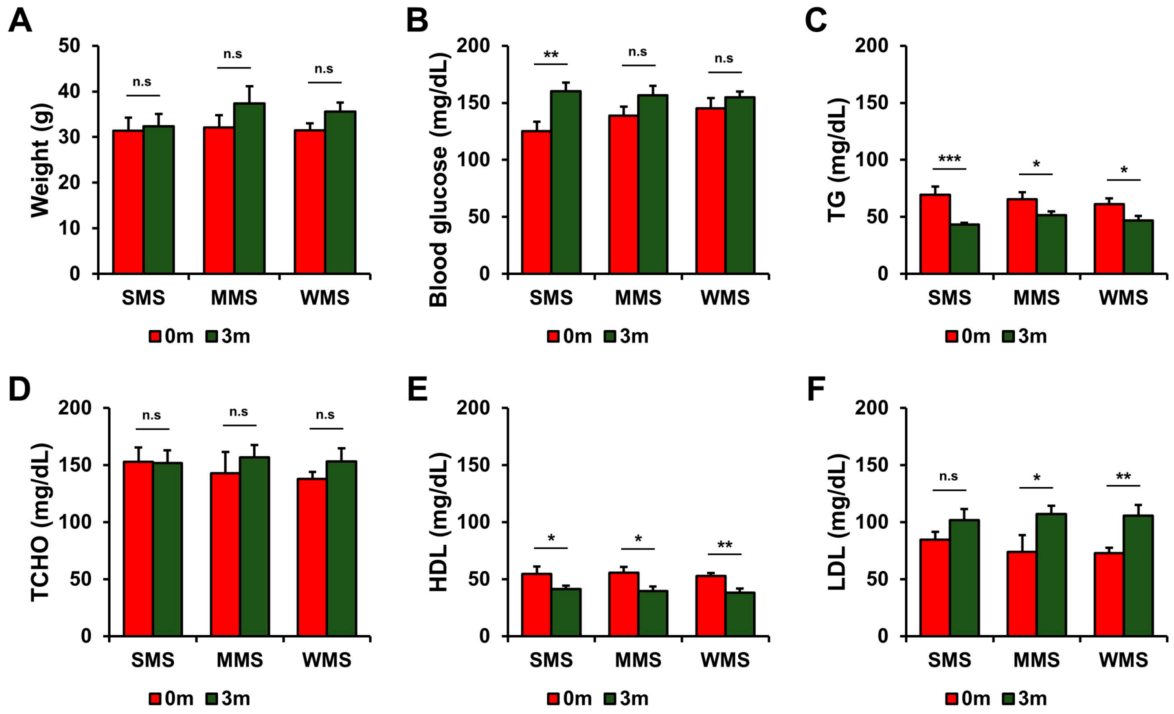

Figure S1. The effects of FMT on changes in blood glucose, blood pressure, and lipid levels. The average values of **(A)** Weight, **(B)** Blood glucose, **(C)** Total glycerol (TG), **(D)** Total cholesterol (TCHO), **(E)** HDL-cholesterol (HDL-CHO), and **(F)** LDL-cholesterol (LDL-CHO) for each group are shown. The values are represented as mean ± SEM. 0m represents before the FMT, and 3m represents 3 months after the FMT. *$p < 0.05$; **$p < 0.01$; ***$p < 0.001$; *n.s*: not significant ($p > 0.05$)

Figure S2

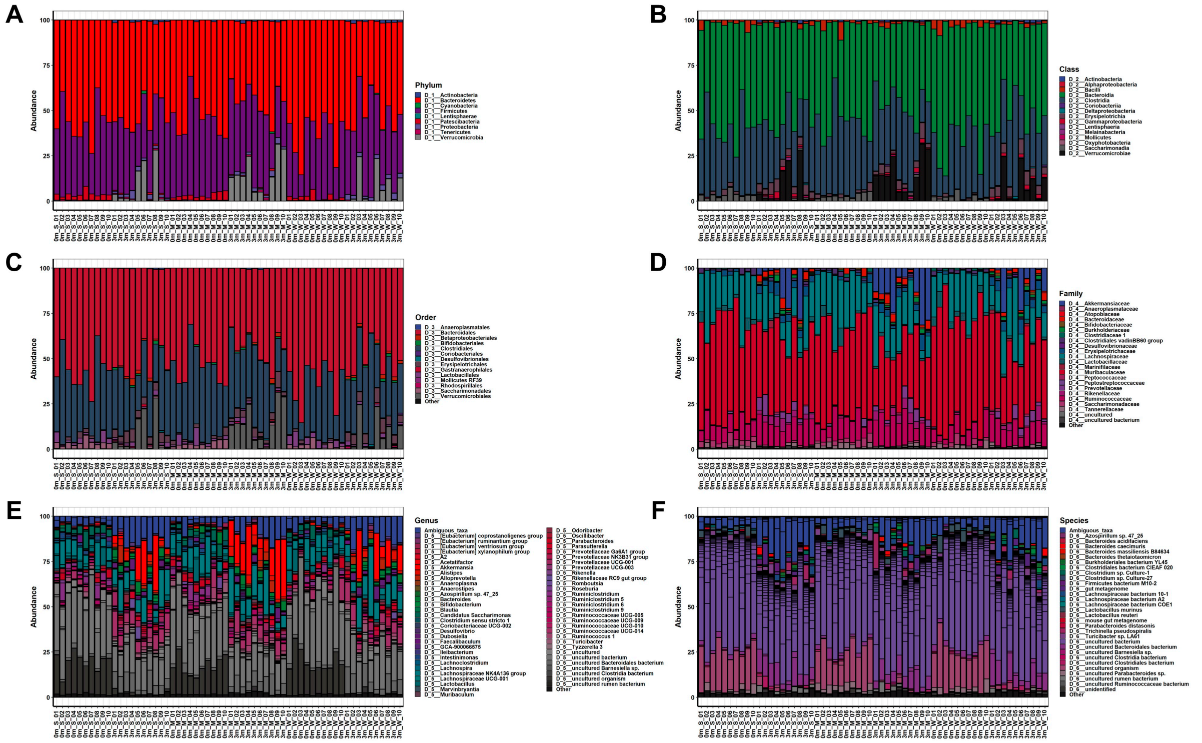

Figure S2. Changes in the composition of the gut microbiome of each mouse before and after FMT. The compositional changes of the gut microbiome at **(A)** phylum level, **(B)** class level, **(C)** order level, **(D)** family level, **(E)** genus, and **(F)** species level are shown. 0m represents before the FMT, and 3m represents 3 months after the FMT. S, M, and W represent the SMS group, MMS group, and WMS group respectively.

Table S1. The valid reads of the 16S rRNA amplicon sequence

Group	0m_S	0m_M	0m_W	3m_S	3m_M	3m_W
Total reads	41,550 ± 1,113	44,773 ± 2,565	46,584 ± 1,215	39,230 ± 2,515	40,207 ± 1,718	36,142 ± 2,031

All values are the mean ± SEM. 0m represents before the FMT, and 3m represents 3 months after the FMT. S, M, and W represent the SMS group, MMS group, and WMS group respectively.

Table S2. Comparison of taxonomy abundance at the phylum level

Phylum	0m_S	0m_M	0m_W	3m_S	3m_M	3m_W	0m	3m
**D_1__Verrucomicrobia**	0	0	0.017	7.552	14.915	8.123	0.006	10.197
**D_1__Bacteroidetes**	56.093	53.922	63.633	52.068	43.928	49.416	57.883	48.471
**D_1__Firmicutes**	40.116	42.569	33.938	36.244	37.628	38.496	38.875	37.456
**D_1__Patescibacteria**	3.240	2.791	2.030	0.510	0.373	0.129	2.687	0.337
**D_1__Actinobacteria**	0.055	0.266	0.074	0.929	0.698	1.093	0.132	0.907
**D_1__Proteobacteria**	0.336	0.335	0.253	1.835	1.671	2.264	0.308	1.924
**D_1__Tenericutes**	0.158	0.115	0.055	0.629	0.574	0.236	0.109	0.480
**D_1__Cyanobacteria**	0.003	0.001	0	0.232	0.210	0.241	0.001	0.228
**D_1__Lentisphaerae**	0	0	0	0.001	0.002	0.002	0	0.002

0m represents before the FMT, and 3m represents 3 months after the FMT. S, M, and W represent the SMS group, MMS group, and WMS group respectively.

Table S3. Comparison of taxonomy abundance at the class level

Class	0m_S	0m_M	0m_W	3m_S	3m_M	3m_W	0m	3m
D_2__Verrucomicrobiae	0	0	0.017	7.552	14.915	8.123	0.006	10.197
D_2__Bacteroidia	56.093	53.922	63.633	52.068	43.928	49.416	57.883	48.471
D_2__Clostridia	36.684	38.674	29.873	30.437	32.948	32.293	35.077	31.893
D_2__Erysipelotrichia	0.943	1.396	0.695	4.538	3.467	4.969	1.011	4.325
D_2__Saccharimonadia	3.240	2.791	2.030	0.510	0.373	0.129	2.687	0.337
D_2__Bacilli	2.489	2.499	3.370	1.269	1.211	1.234	2.786	1.238
D_2__Actinobacteria	0	0.193	0.004	0.735	0.515	0.846	0.066	0.699
D_2__Coriobacteriia	0.055	0.074	0.070	0.194	0.183	0.247	0.066	0.208
D_2__Deltaproteobacteria	0.142	0.193	0.124	0.245	0.306	0.371	0.153	0.308
D_2__Gammaproteobacteria	0.134	0.097	0.089	1.211	1.016	1.438	0.107	1.222
D_2__Mollicutes	0.158	0.115	0.055	0.629	0.574	0.236	0.109	0.480
D_2__Alphaproteobacteria	0.060	0.045	0.039	0.379	0.349	0.455	0.048	0.394
D_2__Melainabacteria	0	0	0	0.230	0.206	0.239	0	0.225
D_2__Oxyphotobacteria	0.003	0.001	0	0.002	0.004	0.002	0.001	0.003
D_2__Lentisphaeria	0	0	0	0.001	0.002	0.002	0	0.002

0m represents before the FMT, and 3m represents 3 months after the FMT. S, M, and W represent the SMS group, MMS group, and WMS group respectively.

Table S4. Comparison of taxonomy abundance at the order level

Order	0m_S	0m_M	0m_W	3m_S	3m_M	3m_W	0m	3m
**D_3__Verrucomicrobiales**	0	0	0.017	7.549	14.915	8.123	0.006	10.196
**D_3__Bacteroidales**	56.093	53.920	63.628	52.024	43.852	49.372	57.881	48.416
**D_3__Clostridiales**	36.684	38.674	29.873	30.437	32.948	32.293	35.077	31.893
**D_3__Erysipelotrichales**	0.943	1.396	0.695	4.538	3.467	4.969	1.011	4.325
**D_3__Saccharimonadales**	3.240	2.791	2.030	0.510	0.373	0.129	2.687	0.337
**D_3__Lactobacillales**	2.489	2.499	3.370	1.267	1.211	1.234	2.786	1.237
**D_3__Bifidobacteriales**	0	0.193	0.004	0.733	0.515	0.846	0.066	0.698
**D_3__Coriobacteriales**	0.055	0.074	0.070	0.194	0.183	0.247	0.066	0.208
**D_3__Desulfovibrionales**	0.139	0.192	0.124	0.245	0.306	0.371	0.152	0.308
**D_3__Betaproteobacteriales**	0.005	0.003	0.005	1.206	1.011	1.432	0.004	1.216
**D_3__Anaeroplasmatales**	0	0.002	0	0.326	0.291	0.071	0.001	0.229
**D_3__Mollicutes RF39**	0.158	0.113	0.055	0.283	0.276	0.160	0.109	0.239
**D_3__Rhodospirillales**	0	0	0	0.366	0.341	0.448	0	0.385
**D_3__Gastranaerophilales**	0	0	0	0.230	0.206	0.239	0	0.225
**Other < 0.5%**	0.195	0.142	0.129	0.092	0.104	0.066	0.155	0.087

0m represents before the FMT, and 3m represents 3 months after the FMT. S, M, and W represent the SMS group, MMS group, and WMS group respectively.

Table S5. Comparison of taxonomy abundance at the family level

Family	0m_S	0m_M	0m_W	3m_S	3m_M	3m_W	0m	3m
**D_4__Akkermansiaceae**	0	0	0.017	7.555	14.923	8.129	0.006	10.202
**D_4__Muribaculaceae**	54.598	50.660	61.670	40.613	33.303	40.784	55.642	38.233
**D_4__Lachnospiraceae**	24.471	24.611	19.667	16.383	18.101	19.080	22.916	17.855
**D_4__Erysipelotrichaceae**	0.944	1.397	0.695	4.542	3.469	4.972	1.012	4.327
**D_4__Saccharimonadaceae**	3.241	2.793	2.030	0.510	0.373	0.129	2.688	0.337
**D_4__Tannerellaceae**	1.259	2.107	1.615	0.653	0.706	0.822	1.661	0.727
**D_4__Lactobacillaceae**	2.466	2.476	3.360	1.249	1.205	1.232	2.767	1.228
**D_4__Ruminococcaceae**	9.720	10.737	7.399	12.561	13.346	11.583	9.285	12.497
**D_4__Prevotellaceae**	0	0	0	4.028	3.089	3.013	0	3.377
**D_4__Bacteroidaceae**	0	0.793	0	2.703	3.156	1.975	0.264	2.611
**D_4__Clostridiales vadinBB60 group**	1.670	1.526	1.391	0.852	0.859	0.798	1.529	0.836
**D_4__Rikenellaceae**	0.253	0.384	0.357	3.431	3.484	2.006	0.331	2.974
**D_4__Bifidobacteriaceae**	0	0.193	0.004	0.733	0.516	0.846	0.066	0.698
**D_4__Atopobiaceae**	0	0	0	0.139	0.118	0.177	0	0.145
**D_4__Desulfovibrionaceae**	0.139	0.193	0.124	0.246	0.306	0.371	0.152	0.308
**D_4__Peptococcaceae**	0.191	0.164	0.154	0.328	0.396	0.533	0.170	0.419
**D_4__Burkholderiaceae**	0.005	0.003	0.005	1.207	1.011	1.433	0.004	1.217
**D_4__Clostridiaceae 1**	0.193	0.710	0.728	0.155	0.079	0.090	0.544	0.108
**D_4__Marinifilaceae**	0	0	0	0.629	0.115	0.790	0	0.511
**D_4__Anaeroplasmataceae**	0	0.002	0	0.326	0.291	0.071	0.001	0.229
**D_4__Peptostreptococcaceae**	0.257	0.761	0.375	0.007	0.004	0.025	0.464	0.012
**D_4__uncultured bacterium**	0.094	0.068	0.038	0.162	0.194	0.083	0.067	0.146
**D_4__uncultured**	0	0	0	0.366	0.341	0.449	0	0.385
**D_4__uncultured bacterium**	0	0	0	0.193	0.183	0.178	0	0.185
**Other < 0.5%**	0.499	0.423	0.371	0.431	0.432	0.429	0.431	0.431

0m represents before the FMT, and 3m represents 3 months after the FMT. S, M, and W represent the SMS group, MMS group, and WMS group respectively.

Table S6. Comparison of taxonomy abundance at the genus level

Genus	0m_S	0m_M	0m_W	3m_S	3m_M	3m_W	0m	3m
D_5__Akkermansia	0	0	0.027	8.155	16.339	8.986	0.009	11.160
Ambiguous_taxa	3.231	3.854	4.155	13.325	8.853	11.553	3.747	11.243
D_5__uncultured organism	18.135	15.826	18.326	0.807	0.426	0.539	17.429	0.591
D_5__uncultured bacterium	30.406	26.769	33.544	15.306	14.418	18.109	30.240	15.944
D_5__Turicibacter	0.828	1.496	0.753	1.994	1.106	1.725	1.026	1.608
D_5__uncultured Bacteroidales bacterium	1.692	1.868	2.096	7.325	6.403	7.555	1.885	7.094
D_5__Lachnospiraceae NK4A136 group	7.477	7.965	7.540	10.866	11.389	12.565	7.661	11.607
D_5__[Eubacterium] coprostanoligenes group	0.027	0.057	0.018	0.681	0.789	0.951	0.034	0.807
D_5__Candidatus Saccharimonas	4.351	3.665	2.690	0.584	0.416	0.147	3.568	0.382
D_5__Parabacteroides	1.681	2.684	2.136	0.753	0.791	0.953	2.167	0.832
D_5__Lactobacillus	3.346	3.125	4.527	1.412	1.339	1.410	3.666	1.387
D_5__Ruminiclostridium	3.731	3.249	1.929	0.624	0.701	0.508	2.970	0.611
D_5__Alloprevotella	0	0	0	1.522	1.517	1.476	0	1.505
D_5__Bacteroides	0	1.033	0	3.044	3.514	2.223	0.344	2.927
D_5__uncultured bacterium	0.987	1.066	0.947	0.475	0.476	0.497	1.000	0.483
D_5__Alistipes	0.336	0.494	0.464	2.548	3.755	1.699	0.432	2.667
D_5__Faecalibaculum	0.419	0.214	0.024	0.375	0.503	0.854	0.219	0.577
D_5__Prevotellaceae UCG-001	0	0	0	1.851	1.353	0.881	0	1.361
D_5__Muribaculum	0	0.072	0	1.069	1.094	1.180	0.024	1.114
D_5__Bifidobacterium	0	0.258	0.005	0.817	0.571	0.967	0.088	0.785
D_5__Intestinimonas	1.744	1.626	0.990	0.186	0.172	0.139	1.453	0.166
D_5__Coriobacteriaceae UCG-002	0	0	0	0.159	0.132	0.197	0	0.163
D_5__Dubosiella	0	0	0.134	2.501	1.866	2.836	0.045	2.401
D_5__Desulfovibrio	0.185	0.247	0.169	0.055	0.069	0.037	0.200	0.054
D_5__Ruminococcaceae UCG-014	1.714	2.268	2.806	7.110	6.026	5.567	2.263	6.234
D_5__uncultured	5.789	6.293	4.524	1.534	1.401	1.578	5.535	1.504
D_5__[Eubacterium] ventriosum group	0.059	0.523	0.222	0.001	0.045	0.004	0.268	0.017
D_5__Oscillibacter	1.119	1.054	0.748	0.551	0.539	0.445	0.974	0.512
D_5__Prevotellaceae NK3B31 group	0	0	0	0.878	0.236	0.662	0	0.592
D_5__[Eubacterium] ruminantium group	0	0	0	0.175	0.176	0.505	0	0.285
D_5__uncultured	0.453	0.601	0.336	0.929	1.164	1.270	0.463	1.121
Ambiguous_taxa	1.030	0.701	0.591	0.261	0.314	0.259	0.774	0.278
D_5__Blautia	0.512	0.400	0.227	0.223	0.401	0.387	0.380	0.337
D_5__Ruminiclostridium 9	1.554	1.383	1.153	1.081	1.302	1.025	1.364	1.136
D_5__Rikenellaceae RC9 gut group	0	0	0	1.256	0.142	0.480	0	0.626
D_5__uncultured	0	0	0	0.153	0.198	0.317	0	0.223
D_5__[Eubacterium] xylanophilum group	0.501	0.502	0.421	0.252	0.321	0.266	0.475	0.280
D_5__Lachnospiraceae UCG-001	1.064	1.030	0.910	0.115	0.205	0.076	1.001	0.132
D_5__uncultured	0.241	0.191	0.183	0.359	0.439	0.589	0.205	0.462
D_5__uncultured Clostridia bacterium	0.109	0.145	0.181	0.020	0.027	0.010	0.145	0.019
D_5__Marvinbryantia	0.383	0.453	0.654	0.101	0.037	0.028	0.496	0.056
D_5__Parasutterella	0	0	0	1.353	1.116	1.603	0	1.357
D_5__Ruminococcaceae UCG-009	0.283	0.345	0.277	0.151	0.192	0.129	0.302	0.157
D_5__Ruminococcus 1	0.008	0.794	0.008	1.032	1.457	1.271	0.270	1.254
D_5__Prevotellaceae Ga6A1 group	0	0	0	0.327	0.093	0.307	0	0.242

**D_5__Ruminiclostridium 6**	0.358	0.331	0.211	0.167	0.395	0.174	0.300	0.245
**D_5__Clostridium sensu stricto 1**	0.259	0.852	0.982	0.107	0.039	0.068	0.698	0.071
**D_5__Roseburia**	0.683	0.584	0.427	0.338	0.350	0.251	0.565	0.313
**D_5__Lachnoclostridium**	0.706	0.535	0.532	0.199	0.348	0.152	0.591	0.233
**D_5__GCA-900066575**	0.744	0.692	0.648	0.123	0.192	0.111	0.695	0.142
**D_5__Odoribacter**	0	0	0	0.656	0.069	0.781	0	0.502
**D_5__Ileibacterium**	0	0	0	0.068	0.186	0.112	0	0.122
**D_5__A2**	0.531	0.410	0.473	0.115	0.086	0.044	0.471	0.082
**D_5__Anaeroplasma**	0	0.003	0	0.369	0.325	0.084	0.001	0.259
**D_5__Lachnospira**	0.268	0.328	0.309	0.010	0.002	0	0.302	0.004
**D_5__Romboutsia**	0.344	1.032	0.503	0.008	0.004	0.030	0.626	0.014
**D_5__Ruminiclostridium 5**	0.417	0.761	0.309	0.159	0.245	0.167	0.496	0.190
**D_5__Anaerostipes**	0	0	0	0.328	0.368	0.251	0	0.315
**D_5__uncultured**	0.006	0.051	0.002	0.105	0.079	0.051	0.020	0.078
**D_5__Ruminococcaceae UCG-010**	0.036	0.065	0.044	0.279	0.184	0.292	0.048	0.252
**D_5__Tyzzerella 3**	0.242	0.231	0.153	0.023	0.024	0.014	0.209	0.020
**D_5__uncultured bacterium**	0.126	0.091	0.053	0.186	0.218	0.099	0.090	0.167
**D_5__Acetatifactor**	0.165	0.148	0.131	0.075	0.122	0.098	0.148	0.098
**D_5__Azospirillum sp. 47_25**	0	0	0	0.250	0.112	0.183	0	0.182
**D_5__Prevotellaceae UCG-003**	0	0	0	0	0.206	0.044	0	0.084
**D_5__uncultured bacterium**	0	0	0	0.209	0.202	0.194	0	0.202
**D_5__uncultured rumen bacterium**	0	0	0	0.070	0.097	0.105	0	0.090
**D_5__Rikenella**	0	0	0	0.077	0.001	0.100	0	0.059
**D_5__uncultured bacterium**	0	0	0	0.078	0.076	0.035	0	0.063
**D_5__Ruminococcaceae UCG-005**	0.032	0.097	0.026	0.026	0.048	0.022	0.051	0.032
**D_5__uncultured Barnesiella sp.**	0	0	0	0.001	0.094	0.002	0	0.032
**Other < 0.5%**	1.686	1.540	1.480	1.674	2.109	1.776	1.569	1.853

0m represents before the FMT, and 3m represents 3 months after the FMT. S, M, and W represent the SMS group, MMS group, and WMS group respectively.

Table S7. Comparison of taxonomy abundance at the species level

Species	0m_S	0m_M	0m_W	3m_S	3m_M	3m_W	0m	3m
D_6__uncultured bacterium	0	0	0.028	8.311	19.514	10.340	0.009	12.722
Ambiguous_taxa	3.708	4.633	4.852	16.394	10.441	13.672	4.398	13.502
D_6__uncultured organism	20.844	19.096	21.358	1.033	0.509	0.633	20.433	0.725
D_6__uncultured bacterium	34.862	32.425	39.085	18.909	17.378	21.369	35.457	19.219
D_6__Turicibacter sp. LA61	0.930	1.765	0.871	2.441	1.323	2.051	1.189	1.938
D_6__uncultured Bacteroidales bacterium	1.954	2.239	2.460	8.975	7.588	8.965	2.218	8.509
D_6__Trichinella pseudospiralis	0	0	0	2.426	3.390	1.644	0	2.487
D_6__Lachnospiraceae bacterium 10-1	0	0	0	1.214	1.071	1.447	0	1.244
D_6__uncultured bacterium	4.979	4.460	3.211	0.712	0.484	0.173	4.217	0.457
D_6__uncultured Parabacteroides sp.	1.955	3.058	2.568	0.012	0.004	0.017	2.527	0.011
D_6__Lactobacillus murinus	2.138	2.014	3.113	0.209	0.473	0.280	2.422	0.321
D_6__uncultured rumen bacterium	0	0.195	0	1.712	1.454	0.841	0.065	1.335
D_6__uncultured bacterium	4.342	3.956	2.314	0.747	0.835	0.585	3.537	0.722
D_6__gut metagenome	0	0	0	1.894	1.716	1.773	0	1.794
D_6__uncultured bacterium	1.149	1.312	1.143	0.579	0.570	0.583	1.201	0.577
D_6__uncultured bacterium	0.004	0.077	0	2.775	3.456	1.464	0.027	2.565
D_6__uncultured bacterium	4.303	4.490	2.540	3.899	4.567	6.337	3.778	4.934
D_6__uncultured bacterium	0.483	0.277	0.026	0.461	0.597	1.004	0.262	0.687
D_6__uncultured bacterium	0	0.092	0	1.326	1.328	1.396	0.031	1.350
D_6__uncultured bacterium	1.984	1.934	1.177	0.101	0.041	0.063	1.698	0.068
D_6__uncultured bacterium	0	0	0	1.784	2.074	3.050	0	2.303
D_6__uncultured bacterium	0.214	0.296	0.200	0.069	0.054	0.028	0.237	0.050
D_6__Clostridiales bacterium CIEAF 020	0	0	0	0.467	0.822	0.813	0	0.701
D_6__Bacteroides caecimuris	0	0	0	0.845	0.436	0.659	0	0.647
D_6__Bacteroides acidifaciens	0	0	0	0.315	0.095	0.078	0	0.163
D_6__uncultured bacterium	0.702	0.832	0.544	0.488	0.322	0.324	0.693	0.378
D_6__uncultured bacterium	0	0	0	1.095	0.287	0.781	0	0.721
D_6__uncultured bacterium	2.870	3.134	2.323	1.021	1.233	1.352	2.775	1.202
D_6__uncultured bacterium	0	0	0	0.229	0.213	0.588	0	0.343
D_6__Clostridium sp. Culture-1	0.176	0.252	0.103	0.870	1.158	1.163	0.177	1.064
Ambiguous_taxa	1.202	0.863	0.719	0.321	0.378	0.306	0.928	0.335
D_6__unidentified	0.242	0.406	0.555	0.320	0.192	0.160	0.401	0.224
D_6__uncultured Bacteroidales bacterium	0	0.149	0	0.639	0.401	0.900	0.050	0.647
D_6__uncultured Clostridiales bacterium	0.527	0.400	0.393	1.255	0.634	0.974	0.440	0.954
Ambiguous_taxa	0	0	0	1.535	0.155	0.543	0	0.744
Ambiguous_taxa	0	0	0	0.184	0.233	0.372	0	0.263
D_6__uncultured bacterium	0.577	0.618	0.504	0.310	0.393	0.312	0.566	0.338
D_6__uncultured bacterium	1.225	1.240	1.068	0.097	0.218	0.058	1.178	0.125
D_6__uncultured organism	0	0	0	0.275	0.332	0.539	0	0.382
D_6__unidentified	0.353	0.273	0.236	0.114	0.160	0.114	0.287	0.129
D_6__Parabacteroides distasonis	0	0	0	0.126	0.338	0.100	0	0.188
D_6__Lachnospiraceae bacterium COE1	0.396	0.331	0.263	0.245	0.239	0.320	0.330	0.268
D_6__uncultured Clostridia bacterium	0.127	0.175	0.220	0.025	0.033	0.012	0.174	0.023
D_6__uncultured bacterium	0.384	0.470	0.692	0.099	0.022	0	0.516	0.040
D_6__uncultured bacterium	0	0	0	0.545	0.357	0.338	0	0.413
Ambiguous_taxa	0.326	0.423	0.329	0.178	0.224	0.138	0.359	0.180
D_6__uncultured bacterium	0.279	0.233	0.220	0.163	0.191	0.169	0.244	0.174

**D_6__Firmicutes bacterium M10-2**	0	0	0.142	1.235	0.062	0.183	0.047	0.493
**Ambiguous_taxa**	0	0	0	0.411	0.107	0.345	0	0.288
**D_6__uncultured bacterium**	0.806	0.720	0.645	0.888	1.143	0.783	0.724	0.938
**D_6__uncultured bacterium**	0.386	0.204	0.317	1.482	1.093	1.099	0.302	1.225
**D_6__uncultured bacterium**	0	0	0	0.164	0.428	0.102	0	0.231
**D_6__uncultured bacterium**	0.296	1.022	1.163	0.129	0.048	0.079	0.827	0.086
**D_6__uncultured bacterium**	0.425	0.363	0.337	0.113	0.101	0.060	0.375	0.091
**D_6__uncultured bacterium**	0.414	0.266	0.362	0.138	0.360	0.106	0.347	0.201
**D_6__uncultured bacterium**	0.452	0.378	0.446	0.114	0.166	0.072	0.425	0.117
**D_6__uncultured bacterium**	0	0	0	0.572	0	0.538	0	0.370
**D_6__Lactobacillus reuteri**	0.207	0.138	0.175	0.144	0.081	0.145	0.173	0.123
**Ambiguous_taxa**	0	0	0	0.080	0.228	0.128	0	0.145
**D_6__Lachnospiraceae bacterium A2**	0.412	0.311	0.360	0	0.003	0	0.361	0.001
**Ambiguous_taxa**	0.238	0.460	0.198	0.102	0.070	0.077	0.298	0.083
**D_6__uncultured bacterium**	0	0.004	0	0.441	0.395	0.100	0.001	0.312
**D_6__uncultured bacterium**	0	0	0	0.165	0.728	0.566	0	0.486
**D_6__Burkholderiales bacterium YL45**	0	0	0	0.794	0.614	1.217	0	0.875
**D_6__uncultured organism**	0	0.324	0.090	0	0	0	0.138	0.000
**D_6__mouse gut metagenome**	0.352	0.264	0.169	0	0	0	0.261	0.000
**Ambiguous_taxa**	0	0	0	0.320	0.310	0.199	0	0.276
**D_6__uncultured bacterium**	0.007	0.064	0.003	0.128	0.093	0.059	0.024	0.093
**D_6__unidentified**	0.212	0.282	0.211	0.038	0.041	0.035	0.235	0.038
**D_6__Bacteroides thetaiotaomicron**	0	0	0	0.085	0.101	0.041	0	0.076
**Ambiguous_taxa**	0	0	0	0.161	0.073	0.372	0	0.202
**D_6__Clostridium sp. Culture-27**	0.140	0.321	0.231	0.026	0.023	0.041	0.231	0.030
**D_6__uncultured bacterium**	0.147	0.107	0.060	0.231	0.252	0.118	0.105	0.200
**Ambiguous_taxa**	0.192	0.181	0.159	0.005	0.001	0	0.178	0.002
**D_6__Azospirillum sp. 47_25**	0	0	0	0.317	0.137	0.216	0	0.223
**D_6__gut metagenome**	0	0	0	0.062	0.382	0.315	0	0.253
**D_6__uncultured Ruminococcaceae bacterium**	0	0.215	0.003	0.058	0.019	0.045	0.073	0.041
**D_6__uncultured bacterium**	0	0	0	0	0.251	0.052	0	0.101
**D_6__uncultured bacterium**	0.203	0.194	0.195	0.032	0.103	0.046	0.197	0.060
**D_6__uncultured bacterium**	0	0	0	0.205	0.219	0.131	0	0.185
**D_6__uncultured rumen bacterium**	0.056	0.017	0	0.054	0.040	0.043	0.025	0.045
**D_6__uncultured bacterium**	0	0	0	0.286	0.231	0.225	0	0.248
**D_6__Bacteroides massiliensis B84634**	0	0	0	0.137	0.519	0.187	0	0.281
**D_6__uncultured rumen bacterium**	0	0	0	0.085	0.115	0.123	0	0.108
**Ambiguous_taxa**	0	0	0	0.099	0	0.117	0	0.072
**D_6__uncultured bacterium**	0	0.040	0	0.084	0.227	0.431	0.013	0.247
**D_6__uncultured bacterium**	0	0	0	0.094	0.091	0.041	0	0.075
**D_6__uncultured bacterium**	0.012	0.026	0.127	0	0	0	0.055	0.000
**D_6__gut metagenome**	0	0	0	0.062	0.101	0.060	0	0.075
**Ambiguous_taxa**	0	0	0	0.015	0.063	0	0	0.026
**Ambiguous_taxa**	0	0	0	0.011	0.259	0.084	0	0.118
**D_6__uncultured Barnesiella sp.**	0	0	0	0.002	0.116	0.002	0	0.040
**D_6__uncultured Clostridiales bacterium**	0	0	0	0.124	0.050	0.009	0	0.061
**Ambiguous_taxa**	0	0	0	0.110	0	0.006	0	0.039
**Other < 0.5%**	1.806	1.983	1.497	2.454	2.625	2.574	1.762	2.551

0m represents before the FMT, and 3m represents 3 months after the FMT. S, M, and W represent the SMS group, MMS group, and WMS group respectively.

Table S8. The α-diversity indexes for each sample and group

Sample	Observed	ACE	Shannon	Simpson	InvSimpson	Fisher
**0m_S_01**	189	189	4.00	0.959	24.51	26.03
**0m_S_02**	217	217	4.44	0.978	44.45	29.98
**0m_S_03**	206	206.15	4.10	0.965	28.67	28.21
**0m_S_04**	208	208	4.02	0.958	23.63	28.25
**0m_S_05**	200	200.19	4.02	0.965	28.61	27.12
**0m_S_06**	210	210	4.26	0.973	37.50	28.51
**0m_S_07**	181	181.21	3.83	0.957	23.27	23.87
**0m_S_08**	236	236.59	4.52	0.979	48.27	33.16
**0m_S_09**	193	193	4.10	0.966	29.60	26.73
**0m_S_10**	199	199.93	4.19	0.967	30.16	27.52
**0m_M_01**	212	212.47	4.29	0.972	36.12	29.26
**0m_M_02**	224	224.25	3.86	0.944	17.86	30.20
**0m_M_03**	197	197.22	4.06	0.969	32.29	27.13
**0m_M_04**	245	245	4.54	0.977	42.60	34.12
**0m_M_05**	265	268.24	4.64	0.983	58.74	37.88
**0m_M_06**	245	245	4.44	0.975	40.33	32.49
**0m_M_07**	223	223.35	4.03	0.958	24.01	29.70
**0m_M_08**	201	201	4.19	0.973	36.42	28.32
**0m_M_09**	194	194.64	4.03	0.966	29.34	26.57
**0m_M_10**	177	177.21	3.85	0.956	22.68	23.57
**0m_W_01**	208	209.31	4.23	0.973	36.46	28.80
**0m_W_02**	171	171	3.61	0.943	17.47	22.31
**0m_W_03**	152	152	3.51	0.949	19.79	19.27
**0m_W_04**	213	213.30	4.29	0.972	35.45	29.21
**0m_W_05**	202	202.51	4.22	0.973	37.19	27.28
**0m_W_06**	204	204.15	3.99	0.961	25.94	27.52
**0m_W_07**	217	217.43	4.16	0.967	29.92	29.26
**0m_W_08**	232	232.76	4.36	0.974	38.29	31.95
**0m_W_09**	162	162	3.64	0.952	20.98	20.68
**0m_W_10**	217	217	4.08	0.964	27.88	29.32
**3m_S_01**	303	303.81	4.46	0.976	41.91	45.01
**3m_S_02**	339	339.73	4.76	0.981	52.98	52.19
**3m_S_03**	410	411.15	4.66	0.972	35.20	62.43
**3m_S_04**	246	249.46	4.28	0.970	33.60	38.53
**3m_S_05**	249	250.11	3.83	0.938	16.08	36.56
**3m_S_06**	418	420.38	4.27	0.957	23.18	61.94
**3m_S_07**	386	386.31	4.61	0.969	32.24	59.04
**3m_S_08**	295	295.16	3.88	0.911	11.21	42.96
**3m_S_09**	377	377.16	4.69	0.977	44.18	56.91
**3m_S_10**	391	396.47	4.74	0.976	42.23	59.53
**3m_M_01**	307	309.10	4.23	0.955	22.34	46.82
**3m_M_02**	291	291.39	4.35	0.964	27.96	43.98
**3m_M_03**	389	389.74	4.54	0.964	27.55	58.11
**3m_M_04**	320	320.16	4.14	0.933	14.87	46.79
**3m_M_05**	402	402.56	5.00	0.987	79.86	62.24
**3m_M_06**	380	380.68	4.81	0.983	58.74	58.89
**3m_M_07**	352	352.17	4.32	0.964	28.07	53.30
**3m_M_08**	318	319.11	3.97	0.946	18.43	47.03
**3m_M_09**	343	344.93	3.58	0.882	8.49	49.63
**3m_M_10**	292	292.94	3.76	0.905	10.55	42.81
**3m_W_01**	313	313.32	4.37	0.969	31.94	47.09
**3m_W_02**	317	318.29	4.32	0.963	26.85	49.32
**3m_W_03**	389	392.35	4.23	0.931	14.54	58.76
**3m_W_04**	388	388.16	4.70	0.975	39.81	58.82
**3m_W_05**	334	334.40	4.07	0.924	13.23	50.21

**3m_W_06**	270	270.76	4.12	0.935	15.28	43.19
**3m_W_07**	291	291.31	4.34	0.969	32.71	43.50
**3m_W_08**	326	326.56	4.43	0.967	29.89	50.19
**3m_W_09**	352	352.16	4.63	0.973	36.62	53.01
**3m_W_10**	294	294.92	4.13	0.956	22.53	43.41

**Group**	**Observed**	**ACE**	**Shannon**	**Simpson**	**InvSimpson**	**Fisher**
**0m_S**	203.9 ± 4.9	204.1 ± 4.9	4.15 ± 0.07	0.967 ± 0.002	31.9 ± 2.8	27.9 ± 0.8
**0m_M**	218.3 ± 8.7	218.8 ± 8.8	4.19 ± 0.09	0.967 ± 0.004	34 ± 3.7	29.9 ± 1.3
**0m_W**	197.8 ± 8.4	198.1 ± 8.5	4.01 ± 0.1	0.963 ± 0.004	28.9 ± 2.5	26.6 ± 1.3
**3m_S**	341.4 ± 20.4	343 ± 20.5	4.42 ± 0.11	0.963 ± 0.007	33.3 ± 4.2	51.5 ± 3.1
**3m_M**	339.4 ± 12.8	340.3 ± 12.7	4.27 ± 0.14	0.948 ± 0.011	29.7 ± 7.1	51 ± 2.1
**3m_W**	327.4 ± 12.5	328.2 ± 12.7	4.33 ± 0.07	0.956 ± 0.006	26.3 ± 3	49.8 ± 1.8

0m represents before the FMT, and 3m represents 3 months after the FMT. S, M, and W represent the SMS group, MMS group, and WMS group respectively.

Table S9. PERMANOVA result tables for group before and after FMT.

Factor	*df*	SS	MS	F	*P*(perm)
**Group (0m)**	2	0.002865	0.0014326	1.1565	0.322
Residuals	27	0.033444	0.0012387		

Factor	*df*	SS	MS	F	*P*(perm)
**Group (3m)**	2	0.0017165	0.0008583	0.99	0.369
Residuals	27	0.0234057	0.0008669		

0m represents before the FMT, and 3m represents 3 months after the FMT. *df*: degrees of freedom; SS: sum of squares; MS: mean sum of squares; F: F value by permutation, *P*(perm): *p*-values based on more than 9000 permutations.

Table S10. Co-occurrence network indices

Network indexes indices	0m_S	0m_M	0m_W	3m_S	3m_M	3m_W
**Node**	73	101	98	204	173	168
**Edge**	65	99	75	158	149	136
**Network Density**	0.025	0.020	0.02	0.008	0.010	0.010
**Assortativity**	-0.019	-0.014	-0.013	-0.007	-0.008	-0.008
**Module**	21	28	31	74	60	58
**Modularity (lc)**	0.879	0.861	0.905	0.961	0.956	0.969

0m represents before the FMT, and 3m represents 3 months after the FMT. S, M, and W represent the SMS group, MMS group, and WMS group respectively.
